# High-flow nasal cannula versus standard low-flow nasal cannula during deep sedation in patients undergoing radiofrequency atrial fibrillation catheter ablation: a single-centre randomised controlled trial

**DOI:** 10.1186/s13063-022-06362-1

**Published:** 2022-05-09

**Authors:** Marloes C. Homberg, Esther A. Bouman, Dominik Linz, Sander M. J. van Kuijk, Bert A. Joosten, Wolfgang F. Buhre

**Affiliations:** 1 Department of Anaesthesiology and Pain Medicine, Maastricht University Medical Centre, Maastricht, the Netherlands; 2 Department of cardiology, Maastricht University Medical Centre and cardiovascular research institute Maastricht, Maastricht, the Netherlands; 3 Department of Cardiology, Radboud University Medical Centre, Nijmegen, the Netherlands; 4 Centre for Heart Rhythm Disorders, Royal Adelaide Hospital, University of Adelaide, Adelaide, Australia; 5 Department of Biomedical Sciences, Faculty of Health and Medical Sciences, University of Copenhagen, Copenhagen, Denmark; 6 Department of Clinical Epidemiology and Medical Technology Assessment, Maastricht University Medical Centre, Maastricht, the Netherlands

**Keywords:** Anaesthesiology, Sedation, Deep sedation, High-flow nasal cannula, Atrial fibrillation, Radiofrequency catheter ablation, Randomised controlled trial

## Abstract

**Background:**

To our knowledge, there are few trials studying the effect of high-flow nasal cannula (HFNC) during deep sedation. Our hypothesis is that high-flow nasal cannula (HFNC) will prevent hypoxemia and desaturation as compared to low-flow nasal cannula (LFNC) during prolonged deep sedation in patients with atrial fibrillation undergoing radiofrequency catheter ablation (RFCA).

**Methods:**

A single-centre, randomised controlled trial with HFNC as the intervention and LFNC as the control group. A total of 94 adult patients per group undergoing elective radiofrequency atrial fibrillation catheter ablation under deep sedation. will be included. The primary outcome is the lowest oxygen saturation (SpO_2_). Secondary outcomes are as follows: the duration of lowest SpO_2_, cross over from oxygen therapy in both directions, incidence of SpO_2_ below 90% > 60 seconds, adverse sedation events, adverse effects of HFNC, mean CO_2_, peak CO_2_ and patients experience with oxygen therapy. The study will take place during the 2-day admission period for RFCA. Patients can fill out their questionnaires in the first week after treatment.

**Discussion:**

HFNC is increasingly used as a technique for oxygen delivery in procedural sedation and analgesia. We hypothesise that HFNC is superior to the standard treatment LFNC in patients under deep sedation with respect to the incidence of desaturation. To our knowledge, there are no adequately powered clinical trial studies on the effects of HFNC in prolonged deep sedation.

**Trial registration:**

ClinicalTrials.gov NCT04842253. Registered on 04 April 2021

## Administrative information

Note: The numbers in curly brackets in this protocol refer to SPIRIT checklist item numbers. The order of the items has been modified to group similar items (see https://nam12.safelinks.protection.outlook.com/?url=http%3A%2F%2Fwww.equator-network.org%2Freporting-guidelines%2Fspirit-2727-statement-defining-standard-protocol-items-for-clinical-trials%2F&data=04%7C01%7C%7Ccebfabce35fe40e5116c08da0ee97648%7C84df9e7fe9f640afb435aaaaaaaaaaaa%7C1%7C0%7C637838693113378492%7CUnknown%7CTWFpbGZsb3d8eyJWIjoiMC4wLjAwMDAiLCJQIjoiV2luMzIiLCJBTiI6Ik1haWwiLCJXVCI6Mn0%3D%7C3000&sdata=aaHdGNuTEzSHq%2FuqF5QPWlcD%2Fk3o7RX5kAxe%2FUb0WIw%3D&reserved=0).

**Table:** 

Title {1}	High-flow nasal cannula versus standard low-flow nasal cannula during deep sedation in patients undergoing radiofrequency atrial fibrillation catheter ablation: A single centre randomised controlled trial.
Trial registration {2a and 2b}.	ClinicalTrials.gov, NCT04842253. Registered on 04 April 2021
Protocol version {3}	First published version: 21 November 2020, version 2
Funding {4}	Support for this study, nasal high-flow equipment and disposable materials and an unrestricted grant, was provided by Fisher & Paykel Healthcare.
Author details {5a}	Marloes C. Homberg^1*^, Esther A. Bouman^1^, Dominik Linz^2,3,4,5,^, Sander M.J. van Kuijk^6^, Bert A. Joosten^1^ and Wolfgang F. Buhre^1^. 1 Department of anaesthesiology and pain medicine, Maastricht University Medical Centre, Maastricht, the Netherlands, 2 Department of cardiology, Maastricht University Medical Centre and cardiovascular research institute Maastricht, Maastricht, the Netherlands, 3 Department of Cardiology, Radboud University Medical Centre, Nijmegen, the Netherlands 4 Centre for Heart Rhythm Disorders, Royal Adelaide Hospital, University of Adelaide, Adelaide, Australia 5 Department of Biomedical Sciences, Faculty of Health and Medical Sciences, University of Copenhagen, Copenhagen, Denmark 6 Department of Clinical Epidemiology and Medical Technology Assessment, Maastricht University Medical Centre, Maastricht, the Netherlands *Corresponding author
Name and contact information for the trial sponsor {5b}	Maastricht University Medical Centre Address: P. Debyelaan 25 6229 HX Maastricht The Netherlands
Role of sponsor {5c}	Funder and sponsors will not influence on study design, collection, management, analysis, and interpretation of data; writing of the report; and the decision to submit the report for publication.

## Introduction

### Background and rationale {6a}

A decrease in in oxygen saturation (SpO_2_) may occur in patients during deep sedation resulting in an elevated risk for perioperative adverse events including hypoxemia [15, 23, 27]. The use of pulse oximetry is important as it can detect hypoxemia early [22]. The incidence of hypoxemia, defined as SpO_2_ below 90%, is approximately 168 per 1000 patients in a general procedural sedation and analgesia population [1]. The clinical significance of episodic desaturations resulting in hypoxemia remains debatable. However, many peri-operative incidents are often short-term and limited in nature but still a predictor of a serious complication resulting in permanent injury [16]. Peri-interventional incidents, e.g. hypoxemia, may be associated with procedure-related and procedure-unrelated complications due to unstable sedation and patient movement potentially injury resulting in permanent injury. Using HFNC during deep sedation is a harmful way to prevent patients from desaturations and hypoxemia and may reduce the risk on perioperative events.

High-flow nasal cannula (HFNC) is increasingly used as a technique for oxygen delivery in patients undergoing procedural sedation and analgesia (PSA) [6]. Humidified HFNC therapy is a form of non-invasive respiratory support. The HFNC technique permits flows up to 70 l/min humidified gas to be delivered to the lungs via a nasal cannula. With the use of HFNC, the amount of oxygen can be titrated more precise in contrast to low-flow nasal cannula (LFNC) [7]. Delivering oxygen at high flow rates has multiple physiological effects that may be beneficial for patients undergoing PSA. First, high inspiratory flow during HFNC increases upper airway pressure resulting in a reduced incidence of upper airway obstruction during sedation [26]. Second, HFNC improves functional residual capacity potentially reducing ventilation-perfusion mismatch [2]. Third, humidification improves mucociliary clearance of secretions which reduces work of breathing [11]. HFNC may prevent the peri-procedural complications typically associated with LFNC.

Several randomised controlled trials have been conducted to test the physiological effects of HFNC [5, 8, 9, 19, 26, 28]. The randomised controlled trials were conducted across a variety of critical care populations including patients with acute respiratory failure [8, 19] or after major surgery [9, 28]. However, adequately powered trials of HFNC in patients undergoing PSA are lacking.

Until now, only three small randomised controlled trials of HFNC in patients undergoing PSA have been published. Sago et al. randomised 30 patients undergoing dental surgery into three treatment groups [26]. Patients received a fraction of inspired oxygen concentration of 40% either via HFNC at a rate of 50 l/min and 30 l/min per minute or via a standard nasal cannula at 4 l/min. Participants randomised to the HFNC groups had higher blood oxygen levels recorded than the low-flow group. Participants who received a flow rate higher than 30 l/min under PSA maintained upper airway patency. Douglas et al. randomised 60 participants undergoing bronchoscopy to receive HFNC at 50 l/min with 100% oxygen or to receive 10–15 litres oxygen per minute with a face mask [5]. Desaturation occurred in 4 out of 30 patients allocated to the HFNC group as compared to 10 out of 30 allocated to the standard oxygen group. No statistical difference for the primary outcome (defined as SpO_2_ < 90%) was found between the treatment groups. Riccio et al. included 59 morbidly obese patients undergoing colonoscopy to receive the same fraction of inspired oxygen concentration of 36% either via HFNO at a flow rate of 60 l/min or via nasal cannula at 4 l per minute [24].

No difference in oxygen saturation and the incidence of desaturation periods was reported. From these studies, it can be concluded that HFNC per se did not result in a substantially improved oxygen status. However, it needs to be noted that this conclusion is based on short procedures requiring PSA with a duration of less than 75 min.

In a number of indications, PSA is applied for longer periods. One of these indications is radiofrequency catheter ablation (RFCA) in AF patients with a duration a PSA of more than 3 h. AF ablation has become one of the most common procedures in the electrophysiology lab with rapidly increasing volumes. A recent survey by the European Heart Rhythm Association (EHRA) shows that in 2019, 32% of all procedures are performed under conscious sedation and 27.5% of all procedures under deep sedation [10]. The most commonly used hypnotic drugs were propofol and midazolam, whereas the most commonly used opioids were remifentanil and fentanyl [10]. The potential positive effects of HFNC in this growing patient population is unclear.

In the Maastricht University Centre patients undergoing RFCA are managed by deep sedation with a combination of low dose remifentanil and propofol, as patient movement may result in shifts of the electro-anatomical maps required for the navigation of the ablation catheter and might cause serious complications including air embolisms and cardiac tamponade. Deep sedation is associated with an increased incidence of upper airway collapsibility and disturbed central respiratory drive [13], which results in forced breathing movements during obstructive respiratory events resulting in pronounced intrathoracic pressure swings leading to electro-anatomical map shifts. Additionally, if LFNC is used (standard of care) a sudden drop of oxygen saturation (SpO_2_ < 90%) occurs in 1.5% of patients [17]. Increased pressure in the upper airway during HFNC might reduce upper airway collapsibility during sedation [21] and facilitates active gas exchange during times of hypoventilation [12] allowing stable and long PSA procedures. Additionally, HFNC may enhance carbon dioxide clearance by an entrained and highly turbulent supra-glottic flow vortices created by high-flow nasal oxygen and cardiogenic oscillations [12]**.**

### Objectives {7}

The aim of this study is to test the hypothesis that oxygen supplementation via HFNC as compared to LFNC can prevent hypoxemia and desaturation in patients with atrial fibrillation undergoing RFCA and prolonged deep sedation of 3 h and more.

### Trial design {8}

**Fig. 1:**
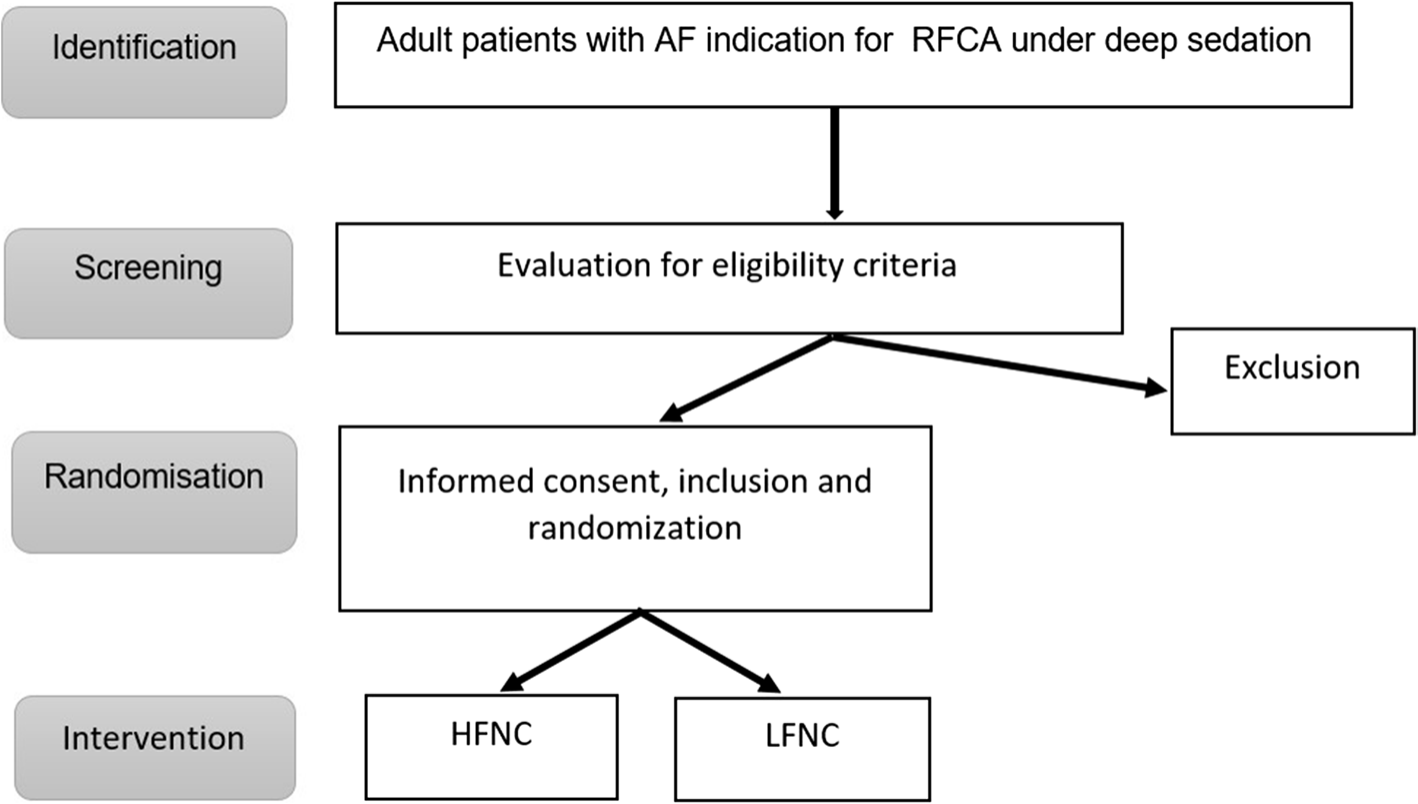
Flow diagram of the study design

This study is a single centre, randomised controlled trial and is designed to evaluate the superiority of HFNC compared with LFNC. Based on a power-calculation {14}, it is aimed to include 188 participants. The allocation ratio is 1:1. Patients will be randomised to the intervention group (HFNC during deep sedation) or the control group (LFNC during deep sedation). Figure 1 summarises the study design.

## Methods: participants, interventions and outcomes

### Study setting {9}

Participants will be recruited at Maastricht University Medical Centre, Maastricht, the Netherlands. Treatment of participants will take place in the same institution.

### Eligibility criteria {10}

#### Inclusion criteria

In order to be eligible to participate in this study, a patient must meet all of the following criteria: Adult (age > 18 years), undergoing an elective RFCA for atrial fibrillation in the MUMC^+^ Cardiac Catheter Labs, under deep sedation administered by a physician assistant (PA) anaesthesiology or nurse anaesthetist (NA)). In this study deep sedation is performed without a protected airway mechanical ventilation.

#### Exclusion criteria

Patients can be excluded of the study if they meet at least one of the following criteria:

- Age under 18 years
- Incapacitated patients
- Body mass index (BMI) > 32 kg/m²
- Diagnosed sleep apnoea syndrome (SAS)
- Chronic pulmonary obstructive disease (COPD) gold IV and COPD gold III with frequent or recent exacerbation
- Diagnosed pulmonary or cardiac condition requiring chronic oxygen therapy
- Complete nasal obstruction
- Active nose bleeding
- Untreated pneumothorax (pre-existing)
- Recent upper airway surgery
- Recent base of skull fracture
- Expected difficult airway
- COVID infection

##### Who will take informed consent? {26a}

The patient receives a written information letter and is additionally informed about the study. At the catheter ablation outpatient clinic, a physician assistant will obtain the informed consent after preoperative screening. The information about the study is explained and the patient has the opportunity to ask questions about the study. After this, the patient is invited to participate in the study. When the participant gives permission for participation, the informed consent form is signed. Patients will receive a copy.

##### Additional consent provisions for collection and use of participant data and biological specimens {26b}

Participants are informed that with signing the informed consent, they give permission for the research team to share relevant data whit regulatory authorities. On the consent form, the participant will be asked if they give permission to store the data for follow-up studies. Participants will also be asked for permission to contact them for future studies. This trial does not involve collecting biological specimens.

### Interventions

#### Explanation for the choice of comparators {6b}

We decided to compare HFNC to a control group with LFNC during deep sedation in patients with atrial fibrillation undergoing radiofrequency catheter ablation. LFNC is standard care during deep sedation [14]. It is expected that HFNC is superior to LFNC during deep sedation.

#### Intervention description {11a}

All randomised patients will receive standard care with regard to medications used for sedation and physiological monitoring or other interventions to support respiratory function that are considered necessary to be initiated during the procedure by the clinicians. Standard care is consistent with the ESA guideline on Procedural Sedation and Analgesia in Adults [14].

Deep sedation is defined as “A drug-induced depression of consciousness during which patients cannot be easily aroused but respond purposefully following repeated or painful stimulation”. Patients may require assistance in maintaining a patent airway, and spontaneous ventilation may be inadequate.

The physician assistant (PA) anaesthesiology or nurse anaesthetist (NA) administers a combination of analgesics and sedatives to target the level of deep sedation. There will be no restrictions of any dose ranges of the different medications used by the PA/NA. The actual doses of medication used will be registered. The PA/NA will record the doses of sedative medications used during procedures.

Oxygen therapy is administrated as indicated by randomisation. The device used for oxygen supplementation will be different.

For HFNC supplemental oxygen will be given with the Optiflow® device (Fisher and Paykel Healthcare, Auckland, New Zealand). The Optiflow® device has a heated breathing tube and chamber, and a nasal cannula will be used. The gas temperature will commence at high settings (37 °C) and titrated downwards if the patient complains of irritation. The gas flow rate will be started at 10 l/min prior to sedation. The gas flow rate of 10 l/min is usually well tolerated by awake patients. The gas flow will be increased after sedation has been administrated to 50 l/min. The gas flow can be titrated according to patients’ requirements. The flow rate is accommodated according to SpO_2_. The FiO_2_ will be set at 40%.

For LFNC, supplemental oxygen will be given with Microstream® Smart CapnoLine® O_2_. Oxygen supplementation is recommended to start at 5 l/min. The flow rate is accommodated according to SpO_2_. The clinician responsible for the sedation will in case of an upper airway obstruction combine the Microstream® Smart CapnoLine® O_2_ with a nasal or oral airway. The use of a nasal or oral Guedel airway is preferred above multiple airway repositioning to protect the clinician against unnecessary exposure to X-rays.

The cardiologic treatment RFCA for atrial fibrillation in combination with the required deep sedation often causes cardiac hemodynamic fluctuations due changes in heart rate. A low dose norepinephrine is used to prevent the occurrence of hypotension. The dose of norepinephrine is titrated to prevent hypotension due to arrhythmias. Temporary pacing is also permitted, due to the nature of the procedure. RFCA and deep sedation are part of the usual medical treatment.

Participants will receive oxygen via a nasal cannula.

There will be no invasive procedures performed. There will be no extra diagnostic procedures performed.

#### Criteria for discontinuing or modifying allocated interventions {11b}

Participants of the study will receive the best medical care.

Protocol deviation is possible in specific cases:

- Cross over from LFNC to HFNC in case of ongoing desaturation. Defined as a saturation < 90% > 180 s and is not improving after airway repositioning, tactile stimulation, increasing the supplemental oxygen or the use of an oral or nasal Guedel airway
- Cross over from HFNC to LFNC in combination with an oral or nasal airway in case of an airway obstruction caused by an upper airway collapse. Also cross over from HFNC to nasal low-flow oxygen if the participant is unable to tolerate the high-flow nasal cannula secondary to discomfort

#### Strategies to improve adherence to interventions {11c}

To improve adherence to the study protocol, the physician assistants (PA) anaesthesiology and nurse anaesthetist (NA) are trained and instructed on the study protocol, use of Optiflow and Sentec Vsign. Data from during sedation will be obtained from the on-line automatic patient data management system, PDMS.

#### Relevant concomitant care permitted or prohibited during the trial {11d}

Participating in this study will not require alternation to usual care pathways (including use of any medication).

#### Provisions for post-trial care {30}

The sponsor has an insurance which is in accordance with the legal requirements in the Netherlands (Article 7 WMO). This insurance provides cover for damage to research subjects through injury or death caused by the study.

### Outcomes {12}

#### Primary outcome

Primary outcome is the lowest SpO_2_. During PSA continuous SpO_2_ measurements will be recorded using SenTec Digital Monitoring with Vsign 2 sensor. Percentage haemoglobin saturated with oxygen will be measured continuously during the procedures as part of routine clinical practice through the anaesthetic monitoring. SpO_2_ monitoring will be measured by the sensor that is placed on the earlobe and recorded continuously.

Timeframe: The time from the first administration of sedative medication to stopping of the sedative medication (= the end of the procedure).

Measure method: Continuous SpO_2_ measurements will be recorded using SenTec Digital Monitoring with Vsign 2 sensor. SpO_2_ will be measured by the Vsign sensor that is placed on the earlobe and will be recorded continuously.

#### Secondary outcomes

- The duration of the lowest SpO_2_

Timeframe: The time from the first administration of sedative medication to stopping of the sedative medication (= the end of the procedure)

Measure method: Duration of the lowest measured SpO_2_ in seconds

- Cross over from oxygen therapy

Timeframe: The time from the first administration of sedative medication to stopping of the sedative medication (= the end of the procedure)

Measure method: cross over yes/no

- To measure the incidence of hypoxemia and desaturation (SpO_2_ under 90% for > 60 s).

Timeframe: The time from the first administration of sedative medication to stopping of the sedative medication (= the end of the procedure)

Measure method: Incidence SpO_2_ under 90% for > 60 s

- The area under the curve of oxygen desaturation (SpO_2_ under 90% for > 60 s).

This is an assembled measure comprising as the difference between threshold of 90% and actual oxygen saturation (SpO_2_) summed every second the duration of oxygen desaturation was longer than 60 seconds

Timeframe: The time from the first administration of sedative medication to stopping of the sedative medication (= the end of the procedure)

Measure method: Difference between SpO_2_ 90% (threshold) for > 60 s and the actual oxygen saturation summed every second during which the oxygen saturation was below threshold

- The incidence of adverse sedation events when oxygen supplementation through HFNC as compared to LFNC in patients with atrial fibrillation undergoing RFCA and deep sedation. The comprehensive research Tool for Tracking and Reporting Outcomes Of Procedural Sedation (TROOPS) is used to register adverse events [25]
  Completion of the tool requires identification and description of the adverse event, the intervention, the outcome and the overall severity of the incident. The PA/NA will be asked to complete this tool at the end of the procedure
- The incidence of adverse effects of delivering high-flow nasal oxygen will be assessed. These adverse effects include pressure injury to the skin caused by the device and nose bleeding due to damage to the mucosal surface. The PA/NA will check the skin integrity around the nasal region at the end of the procedure. The observations will be documented in a case report form provided by the research assistant
- The patient experience with the sedation
  Iowa Satisfaction with Anaesthesia Scale (ISAS) is a validated questionnaire that is used to measure patient experience with the sedation during a procedure [3, 4].
  Timeframe: Before discharge from hospital (day of treatment + 1)
- The Mean TcCO_2_
  Timeframe: The time from the first administration of sedative medication to stopping of the sedative medication (= the end of the procedure)

Measure method: TcCO_2_ will be continuously measured during the whole procedure using SenTec Digital Monitoring with Vsign 2 sensor. TcCO_2_ monitoring provides continuous, accurate (mean bias − 0.1 mmHg) and precise (95 limits of agreements within 6 mmHg) estimates of arterial CO_2_ when the sensor is placed on the earlobe

- The Peak TcCO_2_

Timeframe: The time from the first administration of sedative medication to stopping of the sedative medication (= the end of the procedure)

Measure method: TcCO_2_ will be continuously measured and recorded

- Patients rating of comfort of oxygen delivery
  A 5-point Likert scale will be used to rate the patients comfort of oxygen delivery [18, 20]. Participants will be asked to rate their perceived overall comfort with the oxygen delivery device. A 5-point Likert scale is used with ratings of “very uncomfortable”, “uncomfortable”, “neutral”, “comfortable” and “very comfortable”.

Timeframe: Before discharge from hospital (day of treatment + 1)

- Rating of the physician assistant/nurse anaesthetist of difficulty maintaining oxygenation status
  A 5-point Likert scale will be used to rate the PA/NA difficulty maintaining oxygenation status [3, 4]. The AC will be asked to rate their perceived level of difficulty using a 5-point Likert scale with ratings of “very difficult”, “difficult”, “neutral”, “easy”, “very easy”. If rated “very difficult” or “difficult” the AC specify their answer.

Timeframe: As soon as possible after the end of the procedure

Measure method: PA/NA rating of difficulty maintaining oxygenation status during the procedure using a 5-point Likert scale

- Rating of the physician assistant/nurse anaesthetist of user-friendliness of the oxygen delivery device.
  A 5-point Likert scale will be used to rate the PA/NA of user-friendliness of the oxygen delivery device [3, 4]. The AC will rate their perceived level of difficulty of maintaining oxygenation using a 5-point Likert scale with ratings of “very difficult”, “difficult”, “neutral”, “easy” and “very easy”. If rated “very difficult” or “difficult”, the physician assistant specifies their answer.

Timeframe: As soon as possible after the end of the procedure

Measure method: PA/NA rating of user-friendliness of the oxygen delivery device during the procedure using a 5-point Likert scale

- Rating the satisfaction of cardiologists with catheter stabilisation in relation to sedation
  A 5-point Likert scale will be used to rate the satisfaction of cardiologists with catheter stabilisation in relation to sedation will be rated with a 5-point Likert scale [3, 4]. The cardiologists will rate their perceived level of satisfaction with sedation using a 5-point Likert scale with rating of “very unsatisfied”, “unsatisfied”, “neutral”, “satisfied” and “very satisfied”. If rated “unsatisfied” or “very unsatisfied” the cardiologists specify their answer.

Timeframe: As soon as possible after the end of the procedure

Measure method: Satisfaction of cardiologists with sedation using a 5-point Likert scale

### Participant timeline {13}

**Fig. 2:**
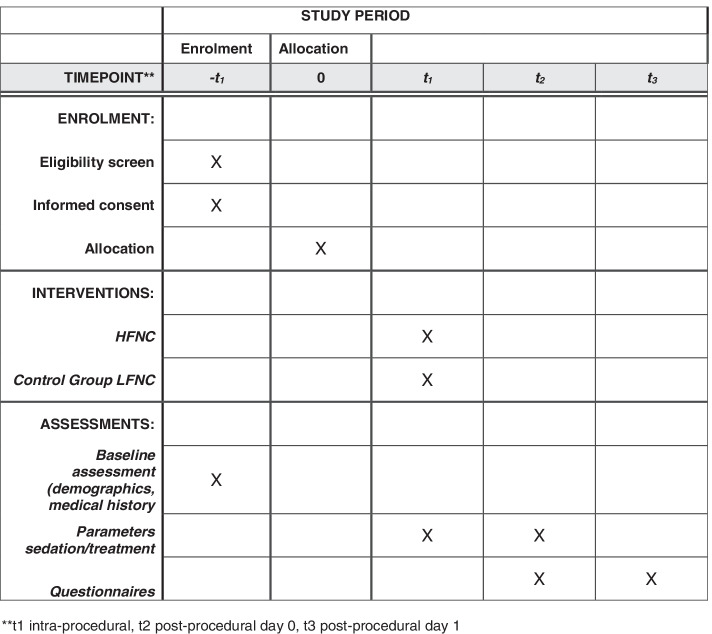
Spirit study schedule of enrolment, interventions and assessments

Participant timeline is summarised in SPIRIT (Fig. 2).

### Sample size {14}

The sample size in this prospective study was calculated according to a similar study with the mean lowest SpO_2_ 95.2% (LFNC) and 99.3% (HFNC), with a standard deviation of 0.1 respectively in two different oxygen therapies [28]. Please note that this sample size is, although based on a similar study which compared the HFNC vs LFNC [8], also based on a different indication and with use of shorter (< 75 min) duration of treatment. To achieve 80% statistical power with α error rate of 0.05, the number of patients was calculated to be 94 for each group. An online statistical calculator, Statulator, was used to calculate the sample size for comparing two independent means.

### Recruitment {15}

The patient will be informed by the cardiologist about the indication for a RFCA treatment during an outpatient clinic visit at the cardiology department. The patient is also informed that treatment will be under deep sedation or general anaesthesia. During this consultation, the patient is asked whether he/she may be approached about current studies. Each patient who indicates to be open to participate in current studies will receive a patient information letter and will be informed by phone about the study aims and risks.

### Assignment of interventions: allocation

#### Sequence generation {16a}

The allocation of participants to groups will be randomised using computer-randomised allocations (data management system used: Castor). Sequence generation will be performed in blocks of 2,4,6 stratified by gender.

#### Concealment mechanism {16b}

Allocation concealment will be guaranteed with sealed opaque envelopes.

#### Implementation {16c}

The allocation sequence will be generated by 2 members of the research team. After screening for eligibility, the patients can be allocated. The patients will be allocated on the day of treatment. The assigned allocation by the enrolling will be communicated to the physician assistant (PA)/nurse anaesthetist (NA) using sealed envelopes. The PA/NA performs the treatment which is assigned to the participant.

### Assignment of interventions: blinding

#### Who will be blinded {17a}

Patients or personnel involved in the trial cannot be blinded to the treatment conditions due to the nature of the intervention. The outcome measurements are objective and are on-line registered in an automatic patient data management system, PDMS. Only data analysts will be blinded.

#### Procedure for unblinding if needed {17b}

The design is open label with only data analysts being blinded therefore unblinding will not occur.

### Data collection and management

#### Plans for assessment and collection of outcomes {18a}

Baseline characteristics of the participants will be collected. Data such as age, gender, height, weight, medical history and ASA-classification, will be obtained from the medical record. Data from during sedation such as heartrate, blood pressure, bispectral index (BIS), duration of PSA and duration of procedure will be obtained from the anaesthesia record (on-line automatic patient data management system, PDMS). The ISAS questionnaires [4] is a validated questionnaire that measures patient experience with monitored anaesthesia care. Patients respond to eleven statements (e.g. I felt pain) by placing a six-choice vertical response column (e.g. “disagree, moderate”).

#### Plans to promote participant retention and complete follow-up {18b}

Patients will receive an email after a week if they didn’t response to the questionnaire invitation. All other data are recorded during admission.

#### Data management {19}

Data management system Castor will be used for data storage and the eCRFs. All research data is stored encrypted in the database so the privacy of the participants is ensured. Members of the research team will have access to the source documents with personally identifiable information. All patients will receive a Castor ID. This Castor ID is composed of any numbers of figures, not based on age, date of birth, name or gender (e.g. 000001, 000002, 000003). The key to the code is safeguarded by the research team in the source document. A monitor and national supervisory authorities e.g. IGJ can access the data to control the research team. The handling of personal data will comply with the EU General Data Protection Regulation and the Dutch Act on Implementation of the General Data Protection Regulation (in Dutch: Uitvoeringswet AVG, UAVG).

#### Confidentiality {27}

The key to the code is safeguarded by the research team in the source document. Without authorization one cannot access the source document. Only the research team has authorization to access the source document with personally identifiable information.

#### Plans for collection, laboratory evaluation and storage of biological specimens for genetic or molecular analysis in this trial/future use {33}

Not applicable because there are no biological specimens in this study.

### Statistical methods

#### Statistical methods for primary and secondary outcomes {20a}

##### Primary outcome

The primary outcome parameter is the lowest SpO_2_. SpO_2_ is a continuous variable and will be presented as mean and standard deviation (SD). Univariable between-group analysis using the independent-samples *t*-test will be performed. To adjust for potentially confounding variables, we will perform multiple linear regression analysis. Covariates will be age, gender, BMI, ASA-classification, duration of RFCA procedure, hypotension and BIS. We will impute missing data using multiple imputation with fully conditional specification. The number of imputations will be set to the percentage of incomplete records. All analyses will be performed according to the intention-to-treat principle. All participants who are randomised are included in the statistical analysis and analysed according to the group originally assigned to, regardless of the treatment received.

##### Secondary outcomes

Cross over of therapy is a binary categorical variable. We will compare the difference in cross-over between groups using Pearson’s chi-squared test. The analysis of the incidence of hypoxemia is performed either with logistic or Poisson regression depending on the distribution. All continuous variables like area under the curve SpO_2_, ISAS score, mean TcCO_2_ and peak TcCO_2_ will be presented as group mean and SD. Univariable analysis using the independent-samples *t*-test will be performed. Further analysis will be done using multiple linear regression. Covariates will be specified as age, gender, BMI, ASA-classification, duration of RFCA procedure, hypotension and BIS. The occurrence of adverse events will be presented as count and percentage per group. Analysis will be performed using a logistic regression model. Likert type questions will be reported as percentage of patients that scored each of the options, stratified by group. The median for central tendency and frequencies for variability are calculated for analysing these descriptive statistics. The response on the Likert type questions will be analysed separately for each question using the Mann-Whitney *U* test.

### Interim analyses {21b}

After inclusion of 50 participants in each group we will perform a blinded sample size control with the SpO_2_ data of the participants. The sample size is based on a similar study which compared the HFNC vs LFNC [8]. However, this study was based on a different indication and conscious sedation instead of prolonged deep sedation.

### Methods for additional analyses (e.g. subgroup analyses) {20b}

Additional analyses are not planned.

### Methods in analysis to handle protocol non-adherence and any statistical methods to handle missing data {20c}

All analyses will be performed according to the intention-to-treat principle. We will impute missing data using multiple imputation with fully conditional specification. The number of imputations will be set to the percentage of incomplete records.

### Plans to give access to the full protocol, participant level-data and statistical code {31c}

In case of audits of health authorities and funder or other researchers needing to access data for scientific purposes, we will give access to the full protocol, participant level-data and statistical code.

### Oversight and monitoring

#### Composition of the coordinating centre and trial steering committee {5d}

All researchers will be a part of the trial management committee. Study coordination will be performed by the Department of Anaesthesiology MUMC+. Data acquisition will be performed by 2 members of the research team. Statistical analysis will be performed by the independent statistician

#### Composition of the data monitoring committee, its role and reporting structure {21a}

Clinical Trial Centre Maastricht (CTCM) designed a data monitoring plan for this study. This study has been classified into the *Negligible* risk category. The monitoring tasks are General and Source Data Verification/Review (SDV/R) related tasks. The monitoring intensity can be adapted during the trial. CTCM is independent from the funder and sponsor.

### Adverse event reporting and harms {22}

#### Adverse events

All adverse events reported spontaneously by the subject or observed by the investigator or his staff will be recorded. For example, an adverse event can also be related to a diagnostic procedure (e.g. contrast allergy during the pre-procedure computer tomography) or to an already existing condition (e.g. decompensation cardio based on atrial fibrillation).

#### Serious adverse events

A SAE is any untoward medical occurrence or effect that results in death, is life threatening (at the time of the event), tamponade, requires prolongation of existing inpatients’ hospitalisation, results in persistent or significant disability or incapacity or any other important medical event that did not result in any of the outcomes listed above due to medical or surgical intervention but could have been based upon appropriate judgement by the investigator.

An elective hospital admission will not be considered as a SAE because the participant is admitted for the treatment. Most SAEs are related to the RFCA procedure not to PSA. Considering the severity of the SAEs, they should be reported. The sponsor will report the SAEs through the web portal *ToetsingOnline* to the accredited METC that approved the protocol, within 7 days of first knowledge for SAEs that result in death or are life threatening followed by a period of maximum of 8 days to complete the initial preliminary report. All other SAEs will be reported within a period of maximum 15 days after the sponsor has first knowledge of the serious adverse events.

The investigator will report all SAEs to the sponsor without undue delay after obtaining knowledge of the events, except for the following SAEs: short-term hypoxemia (SpO_2_ < 90% < 180 s), hypoxemia (SpO_2_ < 90% > 180 s) responding on protocol deviation, unexpected extended recovery time > 8 h, unexpected admission on ICU or CCU and the procedure is terminated.

These SAEs will be reported within a period of maximum 30 days. The period of maximum 15 days may be exceeded. All AEs will be followed until they have abated or until a stable situation has been reached. Depending on the event, follow-up may require additional tests or medical procedures as indicated and/or referral to the general physician or a medical specialist. SAEs need to be reported till end of study within the Netherlands.

#### Frequency and plans for auditing trial conduct {23}

Plans for auditing trial conduct are not included. The independent data safety and monitoring committee will provide an assessment of the patient safety and recommendation to the principle investigator and the researchers about the continuation of this trial. The assessment of the data safety and monitoring committee will be communicated to Clinical Trial Center Maastricht. The Ethical Committee of the MUMC+ will be informed via an annual progress report.

#### Plans for communicating important protocol amendments to relevant parties (e.g. trial participants, ethical committees) {25}

In case of protocol modifications (e.g., changes to outcomes, analyses), we will communicate it to the Ethical Committee and trial registry. Protocol amendments will be reported and documented in the Trial Master File.

#### Dissemination plans {31a}

The sponsor and the investigator agreed to publish the results. This is independent of the results of the study. Publication will take place in accordance with the CCMO guideline regarding publication.

## Discussion

Procedural sedation and analgesia has become a common practice given the increasing demand to relieve anxiety, discomfort and pain during invasive diagnostic and therapeutic procedures. In 2018, the Department of Anaesthesiology at MUMC+ performed 2040 PSA procedures. The majority (*n* = 888) of PSA procedures were performed during RFCA and cardiac electrophysiology testing. Our experience is that as the procedure and PSA time increases saturation more often shows a gradual decrease and desaturations occur more frequent. The aim is to prevent patients for desaturations and hypoxemia. BMI as selection criteria was therefore decreased to 32 kg m^−2^ instead of 35 kg m^−2^. And the PSA (and procedure) time is limited to a maximum of 4 h.

To our knowledge, this is the first study to investigate the effects of HFNC during prolonged deep sedation in patients undergoing RFCA. HFNC is increasingly used as a technique for oxygen delivery in procedural sedation and analgesia The effect of HFNC vs LFNC during sedation has been investigated in several small studies. No difference in primary outcomes of oxygen desaturation was reported. The explanation could be the small sample-sizes and the level of sedation. The intervention could have a positive effect in this study because deep sedation is performed in supine position for a prolonged time up to 4 h.

The sample size in this prospective study was calculated and adjusted using a similar study which compared HFNC vs LFNC in a different population [26]. In this study, participants underwent dental surgery. However, the duration of sedation and treatment were shorter (< 75 min), which will affect primary outcome [26]. Therefore, we decided to perform a sample size control.

The secondary outcomes include ratings of experiences of participants and staff using 5 point Likert scales. Validated questionnaires were preferred, but unfortunately these are not available.

To ensure that all colleagues work according to the study protocol and follow GCP guidelines, education and training is provided. The AC are trained to obtain informed consent. The AC are instructed and trained in the use of the Optiflow and Vsign. They are also trained to provide PSA according to this study protocol during sedation. The group of PAs and AC exists of 7 colleagues.

### Trial status

Trial recruitment is started on 15 November 2022. Recruitment will take approximate two years. This is the first protocol version registered on 21 November 2020. We plan to start recruitment by 1 September 2021 and to complete recruitment by 31 August 2023.

## Data Availability

Data can be obtained by contacting the coordinating researcher. Data will be available by contacting by email the coordinating researcher using details on trial published articles. Email for requests: marloes.homberg@mumc.nl.

## Abbreviations

AF: Atrial fibrillation
CTCM: Clinical Trial Center Maastricht
HFNC: High-flow nasal cannula
LFNC: Low-flow nasal cannula
MUMC+: Maastricht University Medical Center+
NA: Nurse anaesthetist
PA: Physician assistant
PDMS: Patient data management system
RFCA: Radiofrequency catheter ablation
